# Exploring the onset of B_12_ ‐based mutualisms using a recently evolved *Chlamydomonas* auxotroph and B_12_ ‐producing bacteria

**DOI:** 10.1111/1462-2920.16035

**Published:** 2022-05-20

**Authors:** Freddy Bunbury, Evelyne Deery, Andrew P. Sayer, Vaibhav Bhardwaj, Ellen L. Harrison, Martin J. Warren, Alison G. Smith

**Affiliations:** ^1^ Department of Plant Sciences University of Cambridge Downing Street, Cambridge CB2 3EA UK; ^2^ School of Biosciences University of Kent Canterbury, Kent CT2 7NH UK; ^3^ Quadram Institute Bioscience Norwich Research Park Norwich NR4 7UQ UK

## Abstract

Cobalamin (vitamin B_12_) is a cofactor for essential metabolic reactions in multiple eukaryotic taxa, including major primary producers such as algae, and yet only prokaryotes can produce it. Many bacteria can colonize the algal phycosphere, forming stable communities that gain preferential access to photosynthate and in return provide compounds such as B_12_. Extended coexistence can then drive gene loss, leading to greater algal–bacterial interdependence. In this study, we investigate how a recently evolved B_12_‐dependent strain of *Chlamydomonas reinhardtii*, metE7, forms a mutualism with certain bacteria, including the rhizobium *Mesorhizobium loti* and even a strain of the gut bacterium *E. coli* engineered to produce cobalamin. Although metE7 was supported by B_12_ producers, its growth in co‐culture was slower than the B_12_‐independent wild‐type, suggesting that high bacterial B_12_ provision may be necessary to favour B_12_ auxotrophs and their evolution. Moreover, we found that an *E. coli* strain that releases more B_12_ makes a better mutualistic partner, and although this trait may be more costly in isolation, greater B_12_ release provided an advantage in co‐cultures. We hypothesize that, given the right conditions, bacteria that release more B_12_ may be selected for, particularly if they form close interactions with B_12_‐dependent algae.

## Introduction

The study of interactions within microbial communities is garnering increased attention as researchers continue to uncover the extent of microbial interdependence (Gude and Taga, 2019; Gralka *et al*., 2020), which is frequently driven by nutrient exchange. These connections extend to microbes across different domains of life, such as between humans and gut bacteria (Round and Mazmanian, 2009), and plants and their companions in the rhizosphere, including rhizobial bacteria (Udvardi and Poole, 2013) and arbuscular mycorrhizal fungi (Chen *et al*., 2018). Similarly, photosynthetic algae often support a range of heterotrophic bacteria in their phycosphere, a region near the algal cell surface analogous to the rhizosphere (Krohn‐Molt *et al*., 2017; Seymour *et al*., 2017; Kimbrel *et al*., 2019). In return the algae receive specific compounds such as growth factors (Seyedsayamdost *et al*., 2011) or vitamins (Croft *et al*., 2005). For example, the marine alga *Ostreococcus tauri* can support the bacterium *Dinoroseobacter shibae* in co‐culture by providing photosynthate, niacin, biotin and *p*‐aminobenzoic acid, and obtain cobalamin and thiamine in return (Cooper *et al*., 2019). Cobalamin (vitamin B_12_) is a structurally complex cobalt‐containing corrinoid molecule that is of particular interest in algal–bacterial interactions because it is only made by prokaryotes and yet more than half of microalgae require it for growth (Croft *et al*., 2005; Tang *et al*., 2010). B_12_ transfer among species, therefore, has an important role in maintaining community structure and function (Heal *et al*., 2017; Gómez‐Consarnau *et al*., 2018; Sharma *et al*., 2019).

Nutrient amendment experiments of aquatic ecosystems have revealed that B_12_ or B_12_‐producers frequently limit phytoplankton growth (Bertrand *et al*., 2007; Koch *et al*., 2011; Paerl *et al*., 2015; Joglar *et al*., 2020; Barber‐Lluch *et al*., 2021), and laboratory experiments have investigated the effect of B_12_ supply on phytoplankton physiology (Bertrand *et al*., 2012; Heal *et al*., 2019; Koch and Trimborn, 2019; Nef *et al*., 2019). Studies have revealed the effects of B_12_ on the methionine cycle, C1 metabolism, and cell growth and division in *Euglena gracilis* (Shehata and Kempner, 1978; Carell and Seeger, 1980), *Tisochrysis lutea* (Nef *et al*., 2019), *Thalassiosira pseudonana* (Heal *et al*., 2019), and *Chlamydomonas reinhardtii* (Bunbury *et al*., 2020). In these species, B_12_ acts as a cofactor for the enzyme methionine synthase (METH), although some, like *C. reinhardtii*, also encode a B_12_‐independent isoform (METE). It is the presence or absence of the METE isoform that is the best determinant of algal B_12_ dependence (Helliwell, 2017), and while B_12_ dependence is widespread, its complex phylogenetic distribution points to multiple instances of METE gene loss (Helliwell *et al*., 2011; Ellis *et al*., 2017).

There are several possible hypotheses for bacterial B_12_ excretion, ranging from as simple as B_12_ release on bacterial cell death (Haines and Guillard, 1974; Droop, 2007), to B_12_ export systems that may be regulated by algae (Kazamia *et al*., 2012; Grant *et al*., 2014; Cruz‐López and Maske, 2016; Peaudecerf *et al*., 2018). The BtuBFCD complex in Gram‐negative bacteria and the BtuFCD complex in Gram‐positive bacteria are the best characterized prokaryotic systems for B_12_ uptake (Rodionov *et al*., 2003; Degnan *et al*., 2014a), but although there is speculation that many vitamin transporters may be bidirectional, this has not been confirmed for cobalamin (Romine *et al*., 2017). The molecular machinery in algae is also uncertain, although a protein involved in algal B_12_ uptake was identified in the diatoms *Thalassiosira pseudonana* and *Phaeodactylum tricornutum* (Bertrand *et al*., 2012), and subsequently in *C. reinhardtii* (Sayer *et al*. submitted), and B_12_ uptake proteins are now predicted by homology to exist in multiple algal species.

Whatever the mechanism of B_12_ release, its presence in the environment paves the way for the evolution of B_12_ ‘providers’ into ‘cheaters’, that is, organisms that benefit from but do not contribute to the nutrient pool (Morris *et al*., 2012). Partial B_12_ biosynthesis pathways are common in bacterial genomes, indicating the evolutionary benefit of dispensing with this metabolically expensive process (Shelton *et al*., 2019). ‘Leakiness’ of a specific process or metabolite is generally considered to be detrimental but unavoidable (Morris, 2015). However, in the context of a mutualistic cross‐feeding relationship, increased leakiness may prove advantageous under certain circumstances (Stump *et al*., 2018). A hypothesis can therefore be developed, whereby it is only when B_12_‐producing bacteria are closely associated with B_12_‐requirers such as photosynthetic algae that provide something in return, that B_12_ release is evolutionarily favoured.

The unicellular chlorophyte, *C. reinhardtii*, is widely used as a model organism for researching photosynthesis and abiotic stress responses (Sasso *et al*., 2018; Salomé and Merchant, 2019), but it has also been used to study microbial symbiosis (Hom and Murray, 2014; Calatrava *et al*., 2018; Durán *et al*., 2021). Here, we use *C. reinhardtii* and B_12_‐producing bacteria to investigate the inherent capacity for a simple mutualism without previous coevolution. We then investigate how the symbiotic dynamics differ between bacteria and the *C. reinhardtii* wild‐type, which is B_12_‐independent, versus its B_12_‐dependent mutant as an analogy for the commensal–mutualism switch that would occur for algae that evolve B_12_ dependence. Perturbing the mutualistic co‐cultures by nutrient addition reveals how each species responds to the presence of other members of a natural community and to what extent the interaction is regulated. Finally, using two strains of the same species of bacteria that release different amounts of B_12_, we investigate how increased B_12_ release, a disadvantage in axenic cultures, proves advantageous in the presence of B_12_‐dependent algae.

## Experimental procedures

### Strains

The wild‐type *C. reinhardtii* strain used in this work originated from strain 12 of wild‐type 137c. The two other mentioned strains, metE7 and revertant, were derived from this strain by experimental evolution: selection for rapid growth under 1000 ng·L^−1^ B_12_ produced a metE mutant, called S‐type, via insertion of a type II transposable element (Helliwell *et al*., 2015). Subsequent excision of this transposon to repair the wild‐type sequence of METE, produced the B_12_‐independent revertant strain. In one instance, imprecise excision of the transposon left 9 bp behind to produce a genetically stable B_12_‐dependent strain, metE7 (Helliwell *et al*., 2015).

*Mesorhizobium loti*, a soil‐dwelling rhizobium that forms facultative symbiotic relationships with legumes, was used as a B_12_ producer in most co‐culture experiments. Strains MAFF303099, the sequenced strain (Kaneko *et al*., 2000) and ΔbtuF and ΔbluB mutants from the STM library (Shimoda *et al*., 2008) were used. Two *Escherichia coli* strains, ED656 and ED662 (ΔbtuF), were also used as B_12_‐producers. *E. coli* strain ED656 was constructed in a similar fashion to ED741 (Young *et al*., 2021). ED656 was generated from *E. coli* MG1655 by engineering it to contain a set of B_12_ biosynthesis genes. Strain ED656 is MG1655 with (Plac)‐T7RNAP/(T7P)‐cobA‐I‐G‐J‐F‐M‐K‐L‐H‐B‐W‐N‐S‐T‐Q‐J‐D‐bluE‐C‐bluF‐P‐U‐B‐cbiW‐V‐E‐btuR‐R. All the genes were cloned individually in pET3a and then subcloned together using the ‘Link and Lock’ method (Deery *et al*., 2012). ED662 (hereinafter ΔbtuF) was derived from ED656 by insertion of a kanamycin resistance cassette in place of *btuF*. *E. coli* JW0154 ΔbtuF::KmR (Keio collection, *E. coli* Genetic Stock Centre) was used for the construction of ED662 (=ED656 ΔbtuF::KmR) by P1‐mediated transduction (Baba *et al*., 2006). *Salmonella typhimurium* AR3612, a metE and cysG double mutant, was used in bioassays to determine the concentration of B_12_ in samples (Raux *et al*., 1996). *Sinorhizobium meliloti* RM1021, *Rhizobium leguminosarum* bv. viciae 3841 and *Pseudomonas putida* were also initially assessed for B_12_ production and release.

### Algal and bacterial culture conditions

Algal colonies were maintained on Tris‐acetate phosphate (TAP) (The Chlamydomonas Sourcebook) with Kropat's trace elements except for selenium (Kropat *et al*., 2011) + 1000 ng·L^−1^ cyanocobalamin (CAS 68‐19‐9; Millipore‐Sigma) agar (1.5%) in sealed transparent plastic tubes at room temperature and ambient light. Colony transfer by spreading on the agar surface using sterile loops was performed in a laminar flow hood. To initiate liquid cultures, colonies were picked and inoculated into filter‐capped cell culture flasks (NuncTM, ThermoFisher), or 24‐well polystyrene plates (Corning®Costar® Merck) containing TAP or Tris minimal medium (The Chlamydomonas Sourcebook) at less than 60% volume capacity and supplemented with a range of cyanocobalamin concentrations. Cultures were grown under continuous light or a light–dark period of 16 h‐8 h, at 100 μmol·m^−2^·s^−1^, at a temperature of 25°C, with rotational shaking at 120 rpm in an incubator (InforsHTMultitron; Basel, Switzerland).

Bacteria were maintained as glycerol stocks stored at −80°C. To initiate bacterial culture, a small portion of the glycerol stock was removed and spread on LB and TY agar plates incubated at 37°C overnight or 28°C for 3 days for *E. coli* and *M. loti,* respectively. After confirming the lack of contamination by colony morphology, single colonies were picked and inoculated into nunc flasks containing Tris minimal medium with 0.1% glycerol. Axenic bacterial cultures were incubated under the same conditions as algal cultures, as described above.

Co‐cultures of algae and bacteria were initiated by inoculating them into Tris minimal medium from log‐phase axenic cultures after dilution to ensure the optical density of algae and bacteria were roughly equal, except where specified. In some cases, co‐cultures were acclimated for several days before making a dilution based on the algal cell density and starting measurements.

### Algal and bacterial growth measurements

Algal cell density and optical density at 730 nm were measured using a Z2 particle count analyser (Beckman Coulter) with limits of 2.974–9.001 μm, and a FluoStar Optima (BMG labtech) or Thermo Spectronic UV1 spectrophotometer (ThermoFisher), respectively. Colony‐forming units (CFU) of algal cells was determined by 10‐fold serial dilution of a culture aliquot in growth media and spotting 10 μl volumes on TAP + 1000 ng·L^−1^ B_12_ 1.5% agar plates, followed by incubation at 25°C, 20 μmol·m^−2^·s^−1^ of continuous light for 4 days and then counting the number of colonies at 100× magnification. *M. loti* and *E. coli* CFU densities were measured similarly but on TY or LB plates with incubation at 28°C in the dark for 3 days or 37°C overnight, respectively. *E. coli* ED662 (ΔbtuF) was distinguished from ED656 by its kanamycin resistance and hence its ability to grow on plates containing 50 μg·ml^−1^ kanamycin. Single cells for all bacteria were too small to count with a light microscope. When bacteria were grown axenically, optical density was also used as a proxy for cell density with measurements at 600 nm on a FluoStar Optima (BMG labtech) spectrophotometer.

### Vitamin B_12_ quantification

Prior to B_12_ quantification, cultures were separated into fractions. In most cases, 1.15 ml of culture was centrifuged at 10 000 g for 2 min, 1.1 ml of the supernatant (media fraction) was aliquoted into a fresh tube, and 1.1 ml of fresh media was used to resuspend the cell pellet (cell fraction). These aliquots were then boiled for 5 min to release B_12_ into solution and then mixed 1:1 with 2*M9 media. The growth response of a B_12_‐dependent strain of *Salmonella typhimurium* (AR3612) incubated for 16 h at 37°C in this mixture was quantified by measuring optical density at 600 nm (Raux *et al*., 1996). B_12_ concentration was calculated by comparing OD 600 nm of the cultures to a standard curve of known B_12_ concentrations using a fitted 4 parameter logistic model and then reported as mass of B_12_ (in pg) per mL of culture from which the cells or media were separated.

## Results

### B_12_ ‐dependent strain of *C. reinhardtii* takes up B_12_ produced by heterotrophic bacterium

*ChlamydomonasC. reinhardtii* is a model alga in part because it grows well on media containing only inorganic nutrients. However, Helliwell *et al*. (2015) were able to generate a vitamin B_12_‐dependent mutant (hereafter metE7) by experimental evolution in the presence of high concentrations of B_12_. We wanted to test the extent to which B_12_‐producing bacteria could support the growth of metE7 and whether metE7 exerted any control over bacterial B_12_ production. To identify a suitable B_12_‐producer we first chose four soil bacteria (*Mesorhizobium loti* MAFF303099, *Sinorhizobium meliloti* RM1021, *Rhizobium leguminosarum* bv. viciae 3841 and *Pseudomonas putida*), which we hypothesized might co‐occur in the environment with *C. reinhardtii* and could grow under similar conditions. We cultured the strains in minimal medium (TP) with glycerol in a 12 h light‐12 h dark regime. After 6 days of culture, the amount of B_12_ in the media and cell fractions was determined (Fig. S1). In the three rhizobia, the level of B_12_ was roughly equal in both fractions, indicating that a significant proportion of synthesized B_12_ is lost to the surroundings. In *P. putida*, however, which unlike the rhizobial strains encodes the outer membrane B_12_ transporter *btuB*, a substantially smaller portion of detectable B_12_ was in the media. Considering that the proportion of B_12_ in the medium was highest for *M. loti*, as well the fact that stable co‐cultures form between *M. loti* and the *C. reinhardtii* relative, *Lobomonas rostrata* (Kazamia *et al*., 2012), we chose *M. loti* for further axenic and co‐culture experiments.

**Fig. 1:**
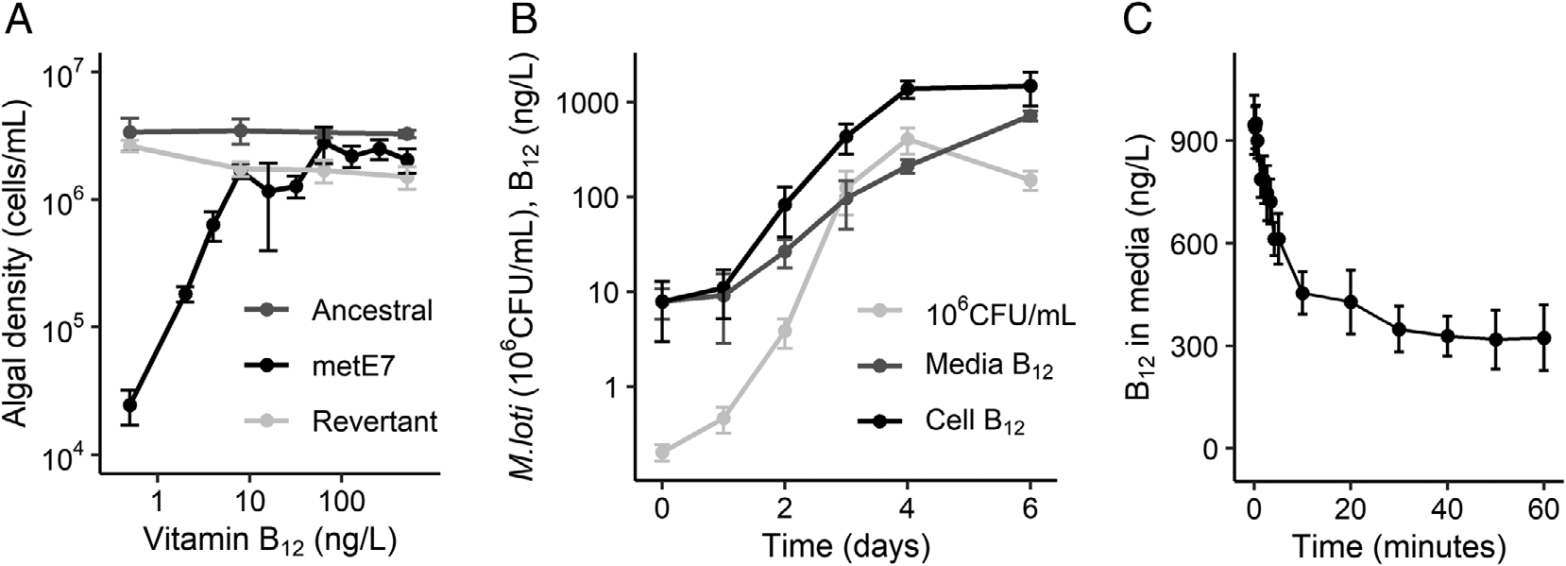
B_12_‐dependent strain of *C. reinhardtii* takes up B_12_ produced by the heterotrophic bacterium *M. loti*. A. The evolved metE7 mutant of *C. reinhardtii*, together with its ancestral line and a revertant (Helliwell *et al*. 2015), were cultured with a range of B_12_ concentrations in TP medium at 25°C with constant illumination at 100 μmol·m^−2^·s^−1^ for 7 days, at which point the cell density in the cultures were measured. B. *M. loti* was cultured in TP media with 0.1% glycerol at 25°C with constant illumination at 100 μmol·m^−2^·s^−1^ and measurements of cell density and B_12_ concentration were made over 6 days. C. An *M. loti* culture, which reached stationary phase, was filtered through a 0.4 μm filter and metE7 cells starved of B_12_ were added to the filtrate at an OD730 nm of 0.1. This culture was then further filtered at multiple time intervals over the course of 1 h to remove metE7 cells and the B_12_ concentration measured in this filtrate. Keys to the different measurements are indicated in legends within the graphs. Error bars represent standard deviations, *n* > = 3.

Before establishing co‐cultures, we quantified the effect of vitamin B_12_ on the growth of the *C. reinhardtii* mutant compared with both the ancestral strain from which it was derived and a revertant strain that was no longer dependent on exogenous cobalamin (see Experimental; Helliwell *et al*., 2015). The three strains were cultured for 7 days with several B_12_ concentrations. As previously demonstrated (Bunbury *et al*., 2020), metE7 maximal cell density showed a clear dose–response to vitamin B_12_, while the growth of the ancestral and revertant strains (both containing a functional METE gene) was not significantly affected by B_12_ (Fig. 1A, Fig. S2). Similarly, the effect of B_12_ supplementation on *M. loti* was quantified for both the wild‐type strain and a B_12_ synthesis mutant (bluB^−^; Shimoda *et al*., 2008), which was confirmed to be unable to synthesize B_12_ (Fig. S3B). Neither strain was affected by B_12_ supplementation (Fig. S3A).

To study the growth and B_12_ production dynamics of *M. loti*, cultures were monitored over a 6‐day period using the same conditions as for *C. reinhardtii*, but with 0.1% (v/v) added glycerol and no added B_12_. Figure 1B illustrates the rapid growth of *M. loti* by more than 1000‐fold within 4 days followed by a small decline by day 6. B_12_ concentration in the bacterial cell fraction increased but to a lesser extent than cell density, suggesting the amount of B_12_ per bacterium decreased, and the B_12_ in the media increased more slowly, but more consistently. To test whether this B_12_ could be used by metE7, we first completely removed all *M. loti* cells by filtering the *M. loti* culture through a 0.4 μm filter and adding the filtrate to a new syringe body. Axenic metE7 in late log‐phase that had been precultured phototrophically with 200 ng·L^−1^ of B_12_ (an amount that is sufficient to avoid B_12_ deprivation while also preventing the accumulation of substantial B_12_ stores) was added to the *M. loti* filtrate at a final OD730 of 0.1. The 1 ml of filtered aliquots was then sampled at predetermined intervals over the next hour from this mixture of *M. loti* filtrate + metE7 to determine how much B_12_ had been taken up by the algal cells. Figure 1C shows that the concentration of B_12_ measured in the filtrate (i.e. B_12_ not taken up by metE7) declined substantially within 20 min, from almost 1000 to 400 ng·L^−1^ with little decline thereafter. This indicates that *M. loti* can produce and release significant quantities of B_12_ and this can be taken up rapidly by metE7. In contrast, we found that *M. loti* is incapable of taking up exogenous B_12_ (Fig. S3), and this may explain why B_12_ progressively accumulates over time in the media fraction of *M. loti* cultures.

### metE7 and *M. loti* support each other in mutualistic co‐culture

**Fig. 2:**
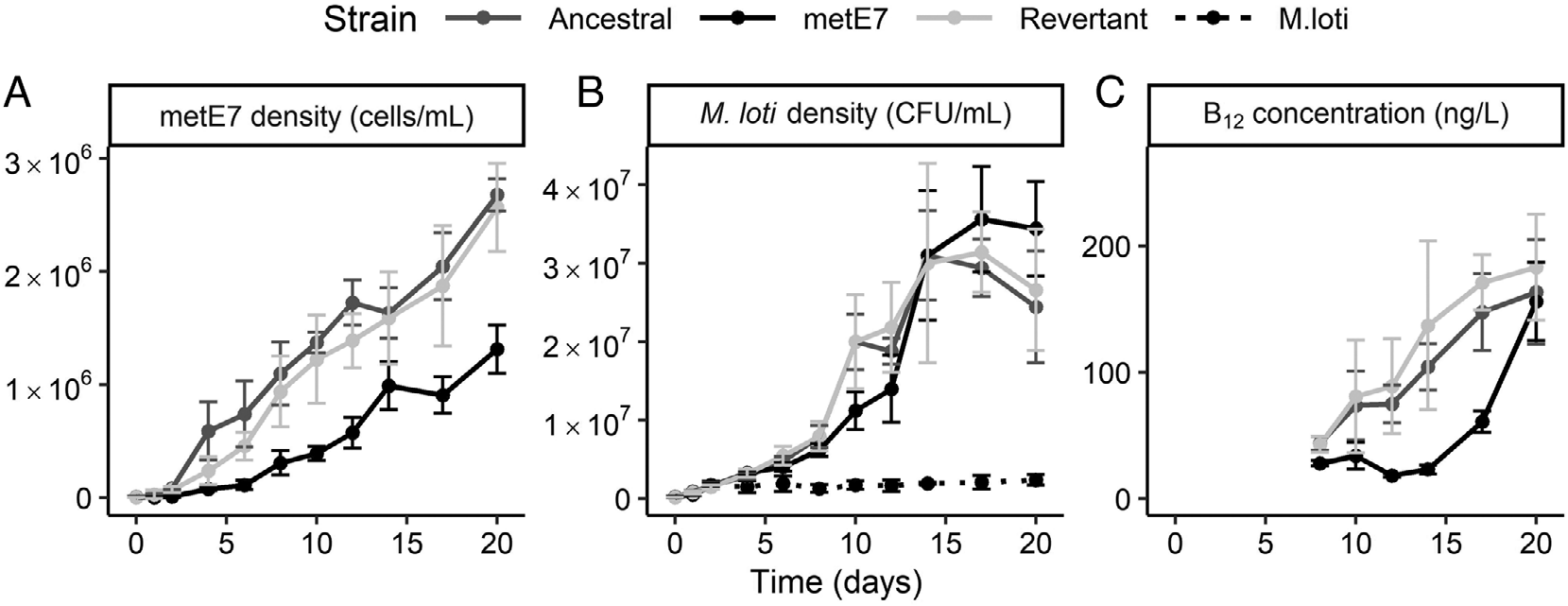
Comparison of commensal and mutualistic co‐cultures between various strains of *C. reinhardtii* and *M. loti*. *M. loti* was co‐cultured with the revertant or ancestral lines or metE7 in TP medium at 25°C and with illumination at 100 μmol·m^−2^·s^−1^ over a 16:8 h light dark cycle for 20 days with periodic measurements of algal and bacterial density as well as B_12_ concentration. A. Measurement of algal density by particle counter; the ancestral and revertant line density increases at a faster rate than metE7 density. B. *M. loti* cell density determined by plating serial dilutions of the cultures on TY media; *M. loti* density is not significantly higher in co‐culture with the ancestral or revertant lines than with metE7 on almost every day. Dotted line indicates axenic growth of *M. loti* in TP medium (i.e. with no *C. reinhardtii* strain). C. Total B_12_ concentration measured by *S. typhimurium* bioassay on aliquots of the co‐cultures (media and cell fraction); B_12_ is generally higher in co‐culture with the ancestral or revertant line. Dark grey = ancestral line, light grey = revertant line, black = metE7. Error bars = standard deviation, *n* = 5.

After confirming metE7 could take up B_12_ produced by *M. loti*, we wanted to see how well *M. loti* would support metE7 in a co‐culture with no B_12_ or exogenous organic carbon, so that *M. loti* would depend on the photosynthate provided by metE7. For comparison with this mutualistic interaction, we also set up two commensal co‐cultures containing the ancestral or revertant strains (both B_12_‐independent) with *M. loti*. Figure 2A shows that the ancestral and revertant strains were able to grow more quickly and to a higher density than metE7 from day 2 onwards (day 20 Tukey test *P* < 0.001 for ancestral and revertant co‐cultures vs metE7 co‐culture), suggesting that low B_12_ levels were limiting metE7 growth. Despite the lower growth of metE7, *M. loti* density was similar in all three co‐cultures (day 20 ANOVA; *P* > 0.05) and significantly higher than the axenic *M. loti* culture (day 20 Tukey test; *P* < 0.001) (Fig. 2B). Nonetheless, the total amounts of B_12_ were significantly lower in the mutualistic than commensal co‐cultures up until the final day, when they equalized (day 20 ANOVA; *P* > 0.05) (Figs. 2C, S4).

Co‐cultures provide some insights into how B_12_ producers and consumers naturally grow and survive in the environment, but ecosystems are clearly more complex due to the presence of multiple species and the fluctuations in physical conditions and nutrient availability. We chose to manipulate nutrient influx in the mutualistic co‐cultures of metE7 and *M. loti* by testing the effect of added glycerol or B_12_. We hypothesized that in the co‐culture, metE7 and *M. loti* were limited by, and so would increase in response to, the addition of organic carbon and B_12_, respectively. We also wished to test whether the non‐requirer would benefit indirectly through the increased growth and nutrient release by its partner. The co‐cultures were initially grown phototrophically as before, with no B_12_ or glycerol, then split into three treatments: no nutrient addition, 200 ng·L^−1^ B_12_, or 0.02% (v/v) glycerol. The cultures were maintained semi‐continuously for 8 days by removing 10% of the culture for daily sampling and replacing it with the same volume of the respective media for each treatment.

**Fig. 3:**
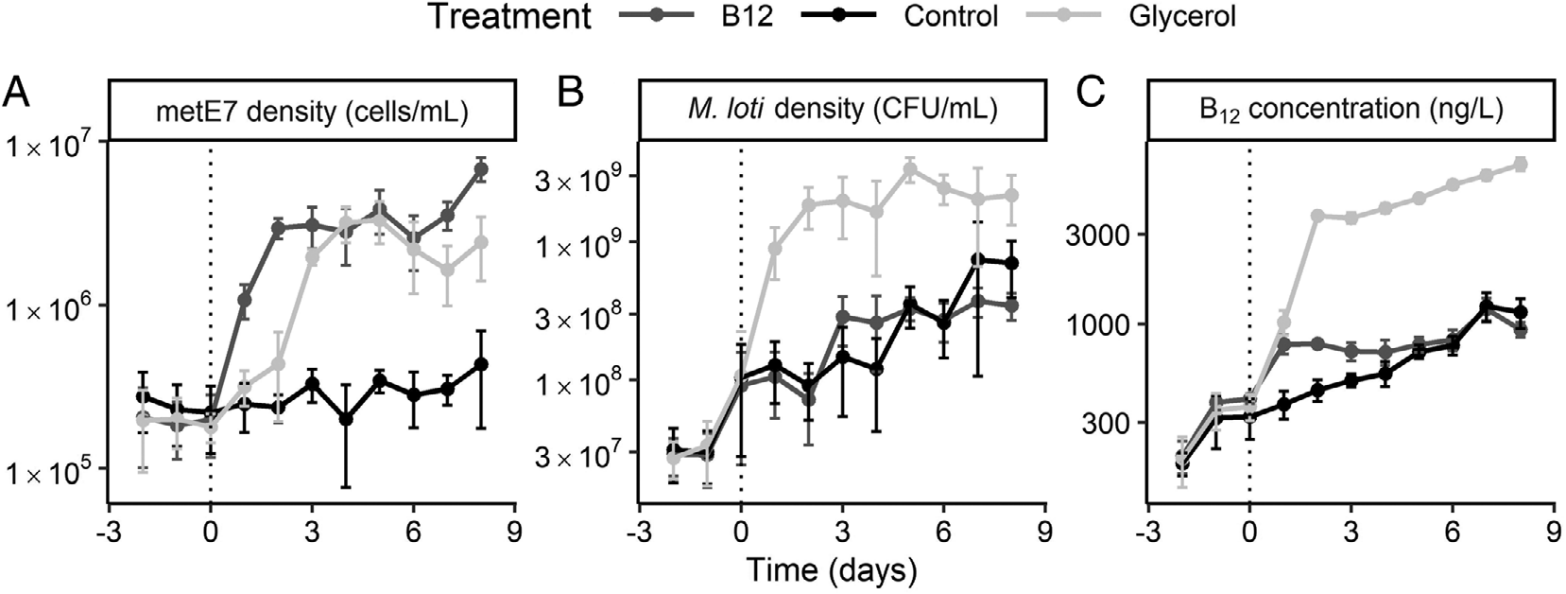
metE7 and *M. loti* only partially support each other's nutrient requirements. *M. loti* and metE7 were cultured semi‐continuously (10% volume replacement per day) under the same conditions as before, but with the addition of 200 ng·L^−1^ of B_12_, 0.02% glycerol or nothing from day 0. Periodic measurements of algal and bacterial density as well as B_12_ concentration in the cells and media were made. A. metE7 density increases in the B_12_ supplemented cultures, but also in the glycerol‐supplemented cultures after a delay. B. *M. loti* density increases in response to glycerol but not B_12_ supplementation. C. Total B_12_ concentration increases more substantially in response to glycerol addition than B_12_ addition itself. Dark grey = 200 ng·L^−1^ of B_12_ addition, light grey = 0.02% glycerol addition, black = control. Error bars = standard deviation, *n* = 4.

As shown in Fig. 3A, the metE7 density increased within 1 day of adding B_12_, but also increased in the cultures that were amended with glycerol, albeit with a delay of 1–2 days. metE7 densities in the glycerol and B_12_‐supplemented cultures appeared to reach a new, roughly stable equilibrium level approximately 10 times higher than the control co‐cultures. On day 8 after nutrient addition, metE7 density was significantly different in each condition (Tukey *P*‐value < 0.05 for all comparisons). Glycerol addition increased *M. loti* levels by ~10‐fold relative to both the control and B_12_‐supplemented cultures (Fig. 3B) (day 8 Tukey *P*‐value > 0.05 for control vs B_12_ and *P* < 0.05 for glycerol vs control or B_12_). However, at no point did the *M. loti* density in the B_12_‐supplemented culture significantly differ from the control, suggesting that the increase in metE7 density did not translate into an increase in organic carbon available for *M. loti* growth. Total B_12_ increased significantly within 1 day of supplementation with either B_12_ itself or glycerol, although by day 8, B_12_ was higher only in the glycerol‐supplemented cultures (Tukey *P*‐value < 0.001) (Fig. 3C). B_12_ in the media, on the other hand, accumulated substantially only after glycerol supplementation (Fig. S5A). Therefore, glycerol addition indirectly increased metE7 density, whereas B_12_‐supplementation, which fuelled the growth of metE7, did not result in increased *M. loti* growth.

A similar study of the symbiosis between the B_12_‐dependent alga *L. rostrata* and *M. loti* found that B_12_ production increased in the presence of *L. rostrata* (Kazamia *et al*., 2012; Grant *et al*., 2014). To determine whether the same was true with metE7 we collected further data from axenic cultures of *M. loti* grown in TP medium with glycerol or co‐cultures of metE7 and *M. loti* in TP medium alone. Figure S6 reveals a clear positive correlation between B_12_ concentration and *M. loti* density, but that B_12_ produced per *M. loti* cell decreases at higher densities. Rather than produce more B_12_ when grown in co‐culture with metE7, *M. loti* produced 45% less B_12_ in total in co‐culture than axenic cultures of a similar density (*P* < 0.0001, Fig. S6A). B_12_ in the media fraction increased less with increasing *M. loti* density in co‐cultures than axenic cultures (*P* < 0.0001; Fig. S6B), presumably due to metE7 B_12_ uptake. B_12_ levels in the cell fraction, conversely, were 50% higher in co‐cultures (metE7 and *M. loti*) than axenic (*M. loti* alone) cultures (*P* < 0.0001; Fig. S6C). Although we could not separate algal and bacterial fractions to measure cellular B_12_ in each, we hypothesized that the reduced B_12_ level in the media of co‐cultures might lead to lower bacterial intracellular levels and hence could relieve suppression on B_12_ riboswitch‐controlled B_12_ biosynthesis operons (Nahvi *et al*., 2004). We therefore tested the effect of removing B_12_ from the media of *M. loti* cultures either by replacing the media entirely or by using metE7 to take up dissolved B_12_. B_12_‐deprived metE7 was capable of absorbing, and so removing, most of the B_12_ released by *M. loti* in a manner that B_12_‐saturated metE7 could not (Fig. S7), and yet there was no subsequent increase in B_12_ production by the bacterial cells under that condition. Similarly, entirely refreshing the media, and so removing all B_12_ from the *M. loti* culture, did not increase B_12_ production. Therefore, it seems unlikely that *M. loti* responds to metE7 B_12_ uptake by increasing B_12_ synthesis.

### Greater bacterial B_12_ release increases both algal and bacterial growth in co‐culture

It was not particularly surprising that metE7 did not increase *M. loti* B_12_ production, since there would be no significant advantage to the naturally B_12_‐independent *C. reinhardtii* to regulate bacterial B_12_ production. However, an evolved B_12_‐dependent alga like metE7 might have a more passive and indirect way of increasing B_12_ in its environment: B_12_‐dependent algae that happen to co‐occur with higher B_12_ providers would grow faster, produce more photosynthate and so improve the growth of the B_12_ producers. To study the effect of B_12_ provision, we first compared the wild‐type strain of *M. loti* with a B_12_ uptake transporter (BtuF) mutant but found no significant difference in B_12_ production or release (Fig. S8). The B_12_‐producing rhizobial strains we had initially tested also did not have substantially different rates of B_12_ release, and due to their different growth rates and different physiologies, determining whether any effect on algal growth was due to B_12_ release would have been challenging. To address this, we took advantage of two strains of *E. coli* engineered to produce B_12_, one of which was further modified so that it lacked BtuF.

**Fig. 4:**
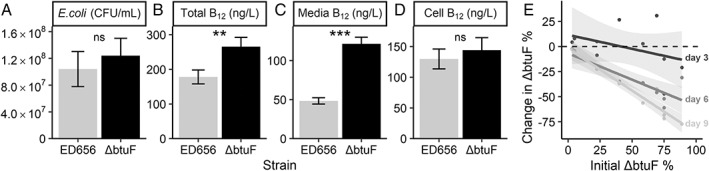
Growth and B_12_ production of two *E. coli* strains engineered to synthesize vitamin B_12_. ED656 expresses BtuF, a protein involved in B_12_ uptake, while ΔbtuF is a kanamycin resistant *btuF* knockout. *E. coli* cells were inoculated at 10^7^ cells·ml^−1^ and grown for 4 days in TP media with 0.1% glycerol (v/v), at 25°C, and with illumination for a 16:8 h light: dark period at 100 μmol·m^−2^·s^−1^. A. *E. coli* density in colony forming units per mL. B_12_ measured by bioassay in the (B) whole culture, (C) supernatant after centrifugation at 10 000 g for 2 min, or (D) pellet after centrifugation. E. *E. coli* strains were grown as above but were initially inoculated at different starting percentages: 4, 20, 50, 80, or 96% ΔbtuF with the remainder made up with ED656. Cultures were maintained by diluting 10 000‐fold on day 3 and 6 after CFU density measurements. CFU density of ΔbtuF and both strains combined were measured over 9 days by plating a dilution series onto LB agar plates with or without kanamycin (50 μg·ml^−1^), respectively, and counting the colonies after an overnight incubation at 37°C. The *x* axis indicates the percentage of *E. coli* that is the ΔbtuF strain on day 0 in each culture, and the *y* axis indicates the change in that percentage on days 3, 6 and 9 (labelled on the right of the plot) compared with day 0. For panels A–D, *n* = 5, for panel E, *n* = 10.

We cultured the two *E. coli* strains, ED656 and △btuF (ED662; see Experimental procedures), in TP media with 0.1% glycerol for 4 days, under the same conditions as were used for *M. loti*, and then measured the cell density by plating on LB plates and B_12_ concentration in various fractions. Figure 4A shows that ED656 and ΔbtuF both grew to a similar density of approximately 10^8^ CFU·ml^−1^, but ΔbtuF produced 50% more B_12_ (*P* < 0.01) (Fig. 4B). Importantly, almost all this additional B_12_ was in the media fraction (Fig. 4C), such that levels were 1.5‐fold higher (*P* < 0.001) for ΔbtuF than ED656, whereas the cellular B_12_ was not significantly different between *E. coli* strains (Fig. 4D). Because the growth rates of ED656 and ΔbtuF were similar, we designed a more sensitive experiment to distinguish them: after mixing both strains in different proportions the cultures were maintained over 9 days under the same conditions as described above with a 10 000‐fold dilution on days 3 and 6. On days 0, 3, 6, and 9 just prior to dilution, the cells were plated on LB plates both with kanamycin and without to determine the numbers of ΔbtuF cells and ΔbtuF+ED656 cells, respectively. The proportion of ΔbtuF cells was substantially lower after 9 days than on day 0 irrespective of the starting concentration, indicating that ED656 had a faster growth rate (Fig. 4E). Since the strains are otherwise isogenic this difference can be attributed to the lack of BtuF, the presence of the kanamycin resistance cassette, or both.

**Fig. 5:**
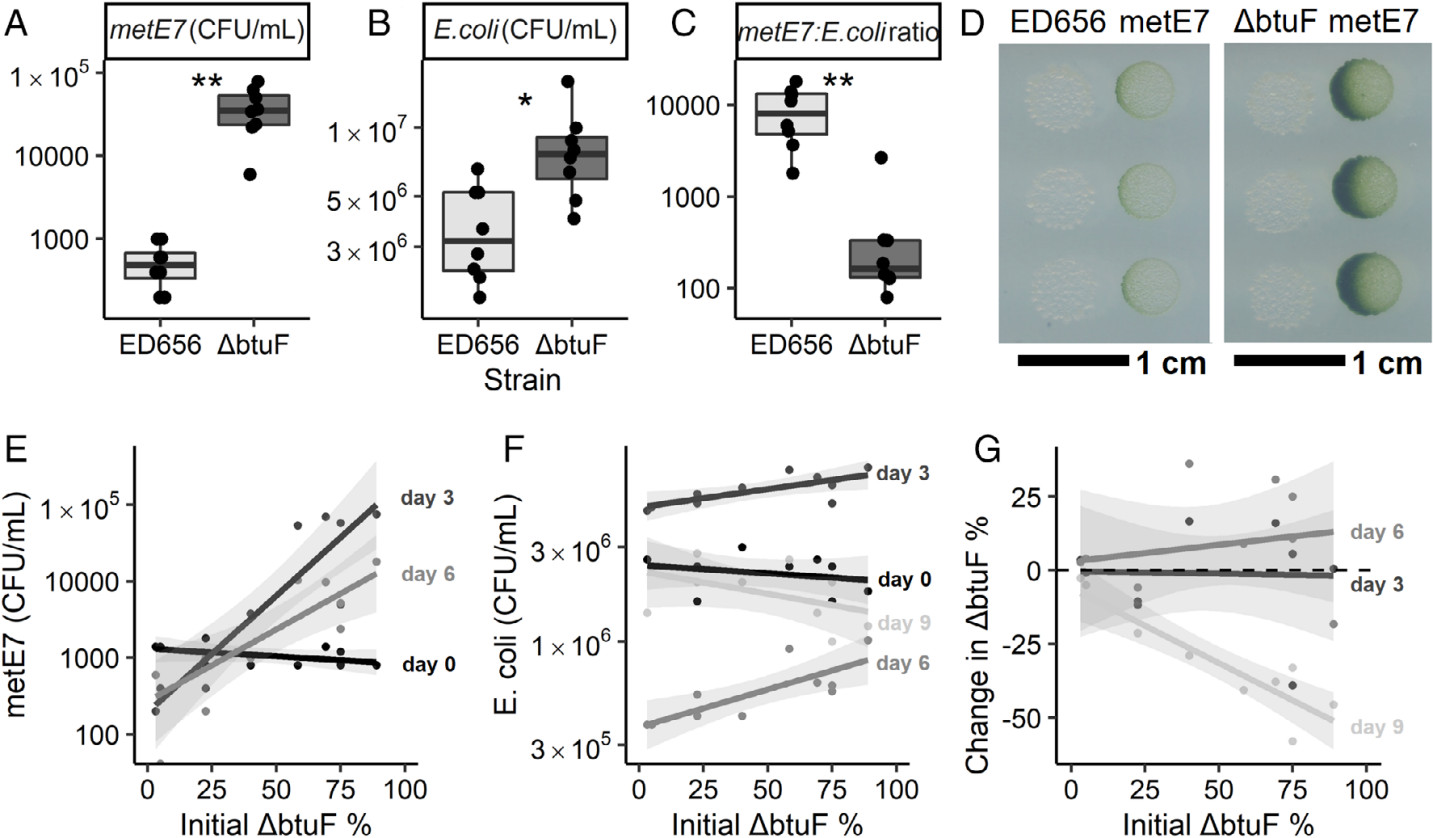
The ΔbtuF mutant of *E. coli* releases more B_12_ and is better than the isogenic parent ED656 at supporting metE7. For panels A–C, metE7 was co‐cultured with either *E. coli* ED656 or ΔbtuF for 3 days at 25°C with shaking at 120 rpm and constant illumination at 100 μmol·m^−2^·s^−1^. A. metE7 colony forming unit (CFU) density on day 2 of co‐culture with either *E. coli* strain. B. ED656 or ΔbtuF CFU density on day three of co‐culture with metE7. C. Ratio of CFUs of *E. coli*:metE7 on day three of co‐culture. D. Photograph of 5 μl droplets of ED656 or ΔbtuF cultures at 10^5^ cells·ml^−1^ spotted 10 mm from metE7 cultures at 10^5^ cells·ml^−1^ on TP agar and incubated for 30 days at 25°C with constant illumination at 100 μmol·m^−2^·s^−1^. For panels E–G, metE7 was co‐cultured for 9 days under the same conditions as above with a mix of ΔbtuF and ED656 strains of *E. coli*, where the intended starting percentage of ΔbtuF in this mix was 4, 20, 50, 80 or 96%. Cultures were maintained by diluting 10‐fold on days 3 and 6 after CFU density measurements. E. metE7 colony forming unit (CFU) density on days 0, 3, and 6 (most metE7 CFU measurements were 0 on day 9). F. Total *E. coli* (ED656 + ΔbtuF) CFU density on days 0, 3, 6, and 9. G. The *x* axis indicates the percentage of *E. coli* that is the ΔbtuF strain on day 0 in each culture, and the *y* axis indicates the change in that percentage on days 3, 6 and 9 (labelled on the right of the plot) compared with day 0. For panels A–C, *n* = 7–8, and for E, F, and G, *n* = 10.

Due to the higher B_12_ production and release by ΔbtuF we predicted that it would support metE7 to a greater extent than ED656. Co‐culturing metE7 and either of the *E. coli* strains in TP media as before revealed that after 3 days metE7 cell density was over 100‐fold greater (*P* = 0.00014) in co‐culture with ΔbtuF than with ED656 (Fig. 5A). This was unlikely to be purely due to increased growth of ΔbtuF, however, because although ΔbtuF grew to a greater extent than ED656, it was only by 1.6‐fold (*P* = 0.046) (Fig. 5B). This resulted in the ratio of bacterial to algal cells for ED656:metE7 being much higher than the ΔbtuF:metE7, at 10 000:1 and 170:1, respectively (*P* = 0.0023) (Fig. 5C). We also tested whether both *E. coli* strains could support metE7 without any physical interaction by spotting them out at equal densities on TP agar 10 mm away from metE7 and incubating them as before. While growth was considerably slower than in liquid co‐cultures, after 30 days, it was visibly clear that both strains had supported growth of metE7, particularly the closest metE7 cells, but that ΔbtuF supported metE7 to a greater extent than ED656 (Fig. 5D).

To further investigate the difference in how well ED656 and ΔbtuF could support metE7, we initiated tricultures, all of which had the same cell density of metE7 and *E. coli*, but which had different percentages of ΔbtuF and ED656 (intended range was 4%–96% ΔbtuF). The densities of each species were determined on days 0, 3, 6, and 9 with 10‐fold dilutions after measurements on days 3 and 6. After 3 days in these tricultures, it was clear that the higher the percent of ΔbtuF the higher the density that metE7 achieved (Fig. 5E; *P* < 0.001). Furthermore, there was a smaller but still significant (*P* < 0.001) positive correlation between percentage ΔbtuF and total bacterial (ΔbtuF + ED656) density (Fig. 5F). However, ΔbtuF was not able to substantially increase as a percentage of the total bacteria (Fig. 5G). By day 9, metE7 levels had collapsed, and ΔbtuF had decreased in prevalence across all starting percentages of ΔbtuF (*P* < 0.01) (Fig. 5G), indicating that ED656 was able to outcompete ΔbtuF in the presence of metE7, as it had done in the purely bacterial co‐culture (Fig. 4E). These results suggest that higher B_12_ releasers could better support B_12_‐dependent algae and benefit themselves in return, but that they would likely nevertheless still be dominated by lower B_12_ releasers in a relatively homogeneous environment.

## Discussion

In this study, we used an experimentally evolved B_12_‐dependent alga to ask how one that arose in the natural environment might survive when supported by B_12_‐producing bacteria. We showed that the B_12_‐dependent mutant of *C. reinhardtii*, metE7 (Helliwell *et al*., 2015), and the rhizobium *M. loti* could support one another's growth (Fig. 2). However, metE7 grew more slowly than the B_12_‐independent ancestral strain in co‐culture with *M. loti*, and B_12_ addition to the co‐culture increased metE7 growth (Fig. 3). Although there are several reports of regulated interactions between algae and bacteria (Seyedsayamdost *et al*., 2011; Amin *et al*., 2015; Dao *et al*., 2020), we found no evidence that *M. loti* produced more B_12_ in response to encountering metE7. Nonetheless, we subsequently found that higher B_12_ providers supported more productive co‐cultures, increasing both algal and bacterial growth, but that they were outcompeted by lower B_12_ providers. We hypothesize that only in more heterogeneous environments, which would allow higher B_12_ providers to colocalize with or attach to B_12_ auxotrophs, would productive mutualisms develop and stabilize.

metE7 was previously shown to be B_12_‐dependent (Helliwell *et al*., 2015, 2016), and *M. loti* known to produce enough B_12_ to support algal B_12_ auxotrophs (Kazamia *et al*., 2012; Helliwell *et al*., 2018; Peaudecerf *et al*., 2018; Laeverenz Schlogelhofer *et al*., 2021), but here we more accurately determined the B_12_ requirements of metE7 grown under different trophic conditions, and revealed the dynamics of *M. loti* B_12_ production and release. metE7 had a significantly lower requirement for B_12_ (EC50 ~ 10 ng·L^−1^) under phototrophic conditions than optimal mixotrophic conditions, presumably due to a slower growth rate and a lower carrying capacity (Fig. S2). This requirement is similar to laboratory cultures and environmental samples of many fresh and saltwater B_12_‐dependent algae, which mostly show half‐saturation constants below 100 ng·L^−1^ (Sañudo‐Wilhelmy *et al*., 2006; Tang *et al*., 2010; Helliwell, 2017). *M. loti* released sufficient B_12_ to raise the concentration in the media to almost 1000 ng·L^−1^ of B_12_ over 6 days of culture in media optimized for *C. reinhardtii*, which is considerably higher than is found in most aquatic or soil environments (Daisley, 1969; Sañudo‐Wilhelmy *et al*., 2012; Barber‐Lluch *et al*., 2021). It should be noted that this is ~100 000‐fold lower than is produced industrially by fermentation using *Propionibacterium shermanii* or *Pseudomonas denitrificans* (Acevedo‐Rocha *et al*., 2019).

There is no known bacterial system for exporting corrinoids, but exogenous B_12_ addition has been shown to reduce bacterial B_12_ release, as has nutrient deprivation, suggesting some degree of regulation (Bonnet *et al*., 2010; Piwowarek *et al*., 2018). Therefore, B_12_ production per cell may have declined as *M. loti* entered nutrient‐limited stationary phase (Fig. S6) or because B_12_ accumulation caused negative feedback on B_12_ synthesis, although Fig. S3 suggests this is unlikely as B_12_ addition did not affect B_12_ production. metE7 absorbed a large quantity of the B_12_ produced by *M. loti* from the medium over a short period (absorption half‐time of 4 min; see Fig. 1C). Previous work has not quantified the dynamics of B_12_ uptake in *C. reinhardtii* at this resolution, but one study found that within 1 day *C. reinhardtii* cells absorbed 12 000 molecules per cell (Fumio Watanabe *et al*., 1991), considerably less than the roughly 300 000 molecules per cell we found were taken up within 1 h. Studies in *Euglena gracilis* discovered uptake of 400 000 molecules of B_12_ per cell within the first minute, which might be explained by the fact that *E. gracilis* has a cell volume that is approximately 20 times greater than *C. reinhardtii* (Shehata and Kempner, 1977; Sarhan *et al*., 1980; Craigie and Cavalier‐Smith, 1982).

In co‐culture with *M. loti*, metE7 grew less well than its ancestral line suggesting that its growth was B_12_‐limited, and yet the growth of *M. loti* was not significantly lower with metE7 than the B_12_‐independent lines for almost the entire growth period (Fig. 2). One potential explanation may be that the B_12_‐limited metE7 cells released (including through cell death) a greater amount of organic carbon or a different spectrum of compounds leading to a greater bacteria:algal ratio. In fact, increased cell size, cell death and an increase in starch and triacylglycerides were all found to occur in metE7 on B_12_ deprivation (Bunbury *et al*., 2020). Figure 3A clearly indicates that the addition of glycerol allows a large increase in *M. loti* growth and subsequently B_12_ production (Fig. 3C), which in turn is the likely cause of the increase in metE7 cell density. Previous studies of algal–bacterial mutualisms have investigated the effect of nutrient addition to co‐cultures and have produced a variety of results. Two studies of the bacterium *D. shibae* co‐cultured with different algae found that adding vitamins B_1_ and B_12_, which were required by the algae, actually improved bacterial growth by a greater amount (Cruz‐López and Maske, 2016; Cooper *et al*., 2019). Our results were more similar to a study of *L. rostrata* and *M. loti*, which found that B_12_ addition improved algal growth with little to no effect on *M. loti* (Kazamia *et al*., 2012; Grant *et al*., 2014). Unlike the *L. rostrata* study, however, we found that after induction of *M. loti* growth by addition of glycerol, algal density subsequently increased, causing the bacterial:algal ratio to return towards pre‐addition levels (Fig. S5).

Across much of the eubacteria, there is a highly conserved regulatory riboswitch element that is found upstream of B_12_ biosynthesis and transport genes (Rodionov *et al*., 2003). In *M. loti*, these elements are found upstream of B_12_ biosynthesis operons, and B_12_ suppresses the expression of some of these genes, likely resulting in reduced B_12_ production. We hypothesized that metE7 could compete for extracellular B_12_ and so indirectly decrease *M. loti* intracellular levels and potentially, therefore, increase B_12_ production. Although the media of dense co‐cultures contained significantly less B_12_ than axenic cultures of *M. loti* with similar densities, this did not result in increased total B_12_ levels (Fig. S6). The only fraction in which B_12_ was higher in co‐culture was the cellular fraction, presumably because in the co‐culture this includes both algal and bacterial cells. The short‐term effects of perturbing *M. loti* cultures similarly did not indicate that either metE7 addition or B_12_ removal increased B_12_ production per *M. loti* cell (Fig. S6). The only way that the addition of metE7 appeared to affect B_12_ production was through increased growth of *M. loti*, presumably through providing a small amount of photosynthate, and this occurred irrespective of whether metE7 absorbed any B_12_ from the media.

After finding that metE7 did not induce *M. loti* to synthesize more B_12_, in contrast to *L. rostrata* (Kazamia *et al*., 2012), it seemed unlikely that *C. reinhardtii* would have evolved any more complex measures to increase B_12_ supply such as partner selection or sanctioning (Leigh, 2010; Chomicki *et al*., 2020). However, we hypothesized that group selection might superficially resemble partner choice, because those bacteria that produced more B_12_ would increase algal growth and so could benefit from increased photosynthate if there was sufficient spatial structure to effectively exclude lower B_12_ producers. A ΔbtuF mutant of *M. loti* did not release more B_12_ than the wild type (Fig. S8) nor did *M. loti* appear to take up B_12_ (Fig. S3). In another rhizobium, *S. meliloti*, it was found that the previously annotated *BtuCDF* genes were actually involved in cobalt uptake, not cobalamin (Cheng *et al*., 2011); however, cobalamin supplementation did appear to increase the growth of *S. meliloti* (Campbell *et al*., 2006). If the homologues in *M. loti* also do not transport cobalamin, this would explain why ΔbtuF showed no difference in B_12_ release. More work will be necessary to test whether this is similar in other rhizobia, and whether the lack of B_12_ uptake predicts the ability to support algal B_12_ auxotrophs.

BtuF in *E. coli* does bind and transport B_12_ (Borths *et al*., 2002), and so we generated a B_12_‐producing strain (ED656) of *E. coli*, and a *btuF* mutant (ΔbtuF) of this strain that released more B_12_ (Fig. 4). Furthermore, ΔbtuF was better able to support metE7 in co‐culture and subsequently grew better itself (Fig. 5). Although ΔbtuF resulted in greater productivity in co‐culture than the parental B_12_‐producing *E. coli* strain (ED656), it also had a slower growth rate than the latter as indicated by its decreasing proportion in co‐cultures with ED656 (Fig. 4E). This suggests that in a homogeneous co‐culture with a B_12_‐dependent alga, ED656 would eventually dominate ΔbtuF and lead to the steady decline and possible collapse of the co‐culture. Indeed this is what we found (Fig. 5E–G).

Generalizing the example mentioned above to an ecological scenario where cooperative strains are at a disadvantage to ‘cheaters’ (those that contribute less or overexploit a public good) is frequently labelled as a ‘tragedy of the commons’ or a ‘prisoner's dilemma’ (Hardin, 2009). These game theory concepts describe how it can be optimal at the group level for individuals to cooperate while simultaneously being optimal for each individual to cheat, such that the rational outcome (Nash equilibrium) is also the worst outcome overall (Nash, 1950). The fact that mutualistic interactions nevertheless abound in nature indicates that this dilemma is solvable, and spatial structure is often at the heart of these solutions (Stump *et al*., 2018). In multicellular hosts with microbial symbiotic partners, spatial structure is often manifested as compartmentalization, allowing hosts to control, sanction, or reward their endosymbionts (Chomicki *et al*., 2020). In aquatic microbial symbioses, it is the phycosphere of separate algal cells that can provide spatial structure for the stable establishment of different bacterial communities (Kimbrel *et al*., 2019; Durán *et al*., 2022), and vertical transmission (which favours mutualism) (Crespi, 2001) of bacteria to algal daughter cells might even be more common than in plant–rhizobia symbioses.

In our example of an algal B_12_ auxotroph, whether the alga and its associated community survive is at least partially dependent upon whether those bacteria provide sufficient B_12_. It is therefore reasonable to hypothesize that bacteria that release more B_12_, such as the ΔbtuF mutant discussed here, could out‐compete those that release less, only if they associate with algal B_12_ auxotrophs such as metE7 in a manner that spatially excludes the lower producers. There may be similar advantages of enhanced B_12_‐producers feeding B_12_‐auxotrophs in more complex microbial communities, such as those found within the gastrointestinal microbiome, where B_12_ has been shown to be a key modulator of the ecosystem (Degnan et al., 2014b). However, the situation is further complicated by the observation that bacteria produce a range of non‐cobalamin corrinoids (Bryant *et al*., 2020), which may either help specific community formation or prevent predation. Intriguingly, algae, like humans, prefer to utilize cobalamin over other corrinoids (Helliwell *et al*., 2016).

In summary, our results suggest that B_12_‐producing bacteria could support newly evolved algal B_12_ auxotrophs but not necessarily that they would favour the growth of B_12_ auxotrophs over their B_12_‐independent relatives. What precise circumstances drive the evolution of algal B_12_ auxotrophy are therefore still unclear, but complex natural communities may well be more propitious environments for B_12_ auxotrophy than the purely bipartite, reciprocal relationships studied here. Furthermore, other B_12_ auxotrophs may naturally have lower B_12_ requirements than metE7, or evolve lower requirements over time, and likely exist in environments where multiple nutrients colimit growth. Finally, natural selection tends to remove species that produce metabolites in excess of their own needs, and so we propose that only in a more structured environment, including bacterial attachment to algae, might it be beneficial for a bacterium to produce and release more B_12_ rather than compete for its uptake.

## Author contributions

F.B. and A.G.S conceived and designed the research and drafted the manuscript. F.B., E.D. A.P.S, V.B. and E.L.H. participated in data acquisition and analysis. M.J.W. and E.D. contributed resources. All authors helped draft and critically revised the manuscript and gave final approval for publication and agree to be held accountable for the work performed therein.

## Supporting information


**Supplementary Fig. 1.** Vitamin B_12_ levels in the cell and media fraction of axenic cultures of four B12‐producing bacterial strains. Bacteria were grown in TP medium +0.1% glycerol with illumination in a 12:12 h light:dark period at 100 μmol·m^−2^ ·s^−1^ and 25°C with rotational shaking at 120 rpm. After 6 days of growth, cultures were collected, centrifuged, and the pellet (cell fraction) and supernatant (medium fraction) separated and their B12 content measured. The B12 concentrations, in ng/L, are displayed as boxplots for (A) *M*. *loti*, (B) *S*. meliloti, (C) *R*. *leguminosarum*, and (D) *P*. *putida*. *n* = 4 biological replicates.
**Supplementary Fig. 2**. Assessing the B12 dependence of three lines of C. reinhardtii under different trophic conditions. The three lines include the ‘ancestral’ line prior to experimental evolution, ‘metE7’, a stable B12‐dependent line, and ‘revertant’, a B12 independent line that had reverted from a B12‐ dependent line. Cultures were grown heterotrophically (TAP medium in the dark), mixotrophically (TAP medium in continuous light), and photoautotrophically (Tris minimal medium in continuous light). B12 concentrations ranged from 0.5 to 512 ng·L^−1^ and precultures of the algae, which were grown with 200 ng·L^−1^ B12, were washed thrice and inoculated at a density of roughly 100 cells·ml^−1^ . (A) Cell density was measured by particle counter after 6 days of growth for mixotrophic cultures or 8 days for heterotrophic and photoautotrophic conditions. (B) Estimated maximal density of metE7 at unlimiting B12 concentrations calculated by fitting a Monod equation to data in panel A. (C) Estimated concentration of B12 required to produce half the maximal density of metE7 cells under each trophic condition calculated by fitting a Monod equation to data in panel A. *n* = 3–4, error bars = sd.
**Supplementary Fig. 3.** Growth and B12 uptake of M. loti strains. The wildtype (MAFF303099) and B12 synthesis (BluB) mutant were grown in Tris minimal medium supplemented with 0.1% glycerol at 100 μmol·m^−2^ ·s^−1^, and at a temperature of 25°C, with rotational shaking at 120 rpm over a period of 6 days with (1000 ng·L^−1^) or without added B12. (A) Viable cells (colony forming units) of *M. loti* 303 099 increased more quickly than the BluB mutant, but there was no significant effect of B12 on growth rate of either strain. (B) The addition of B12 had no effect on the B12 recovered in the cell fraction (top panel) indicating no B12 uptake. Instead, all the added B12 remained in the media (middle panel). Red lines = 1000 ng·L^−1^ of added B12, Blue lines = no added B12, Error bars = sd, *n* = 4.
**Supplementary Fig. 4**. Dynamics of the ratios of B12, bacterial density and algal density during co‐cultures of *M. loti* and three strains of *C. reinhardtii* (A) B12 concentration expressed as molecules of B12 per algal cell reveal very similar levels although different dynamics for the three *C. reinhardti*i strains (B) B12 concentration expressed as molecules of B12 per M. loti cell reveal lower production in co‐culture with metE7 particularly around day 14 of co‐culture. (C) Bacteria:algae ratio was consistently higher in the metE7 co‐culture. Error bars = sd, *n* = 5.
**Supplementary Fig. 5**. Dynamics of B12 concentrations in the cellular and media fractions and bacteria:algae ratio in metE7 + *M. loti* co‐cultures perturbed by nutrient addition. (A) B12 concentration in the media of co‐cultures reveals that the highest levels were found following addition of glycerol. (B) B12 concentration in the cellular fraction reveals that glycerol addition caused significantly higher B12 production. (C) Bacteria: algae ratio initially diverged after addition of glycerol or B12 followed by a smaller convergence. Error bars = sd, *n* = 4.
**Supplementary Fig. 6**. *M. loti* does not increase B12 production in the presence of metE7. Several axenic cultures of *M. loti* with supplemented glycerol and co‐cultures containing *M. loti* and metE7 (without glycerol) were grown in TP medium at 25°C with illumination at 100 μmol·m^−2^ ·s^−1^ over a 16:8 h light: dark cycle for up to 32 days or up until the cultures crashed. B12 measurements of the media and cell fraction were made periodically (A) Total B12 is higher in axenic *M. loti* culture than co‐culture at the same *M. loti* density (*P* < 0.001) (B) B12 in the media is significantly lower in co‐cultures than axenic cultures at high *M. loti* densities (*P* < 0.001). Grey = *M. loti* axenic culture, black = metE7 + *M. loti* co‐culture. N (axenic) = 106, N (co‐culture) = 284, grey shaded region = 95% confidence interval.
**Supplementary Fig. 7**. B12 production by *M. loti* following removal of B12 from the culture media. (A) Experimental setup: Two sets of axenic *M. loti* cultures (grey) were inoculated with metE7 cells that were either saturated with (black solid) or starved (black dashed) of B12 and incubated for 1 h. All 4 cultures were then passed through a 5 μm filter, removing all metE7 cells but not *M. loti*. These *M. loti* cultures were centrifuged, and the supernatant replaced with fresh Tris‐min media in treatment ‘washed’ (grey dashed), or otherwise resuspended without replacing the supernatant (grey solid). The resuspended, newly axenic *M. loti* cultures were grown for 3 days with illumination in a 16:8 h period at 100 μmol·m^−2^ ·s^−1^ and 25°C with rotational shaking at 120 rpm. (B) Total B12 concentration in the culture, and (C) Total B12 per *M. loti* cell. (D) B12 concentration in the supernatant after centrifuging an aliquot of the sample, and (E) media B12 per *M. loti* cell. (F) B12 concentration in the cell pellet after centrifuging an aliquot of the sample, and (G) cell B12 per *M. loti* cell. Error bars = sd, *n* = 4
**Supplementary Fig. 8**. Growth and B_12_ release of *M. loti* strains. The wildtype (MAFF303099) and B12 transporter mutant (*btuF*) were grown in Tris minimal medium supplemented with various concentrations of glycerol at 100 μmol·m^−2^ ·s^−1^, and at a temperature of 25°C with rotational shaking at 120 rpm over a period of 8 days. (A) Viable cells (colony forming units) of *M. loti* MAFF 303099 increased over time at the same rate as the *M*. *loti btuF* mutant and both strains showed improved growth on increasing the glycerol concentration from 0.0128% (v/v) to 0.0512%, but not with a higher concentration. (B) The amount of B_12_ produced in the cells (top panel) and released into the media (middle panel) were not significantly different in the two strains, but as with the cell growth, did increase with the two higher glycerol concentrations. Blue lines = wildtype (MAFF303099), Blue lines = btuF mutant, Error bars = sd, *n* = 4.
