# Supplementary material for: Exploring the onset of B12 ‐based mutualisms using a recently evolved Chlamydomonas auxotroph and B12 ‐producing bacteria
Source: Environ Microbiol. 2022 May 20;24(7):3134–47. doi: 10.1111/1462-2920.16035 (PMC9545926; doi:10.1111/1462-2920.16035)
Supplement: Supplementary file 1 — Supplementary Fig. 1. Vitamin B12 levels in the cell and media fraction of axenic cultures of four B12‐producing bacterial strains. Bacteria were grown in TP medium +0.1% glycerol with illumination in a 12:12 h light:dark period at 100 μmol·m−2 ·s−1 and 25°C with rotational shaking at 120 rpm. After 6 days of growth, cultures were collected, centrifuged, and the pellet (cell fraction) and supernatant (medium fraction) separated and their B12 content measured. The B12 concentrations, in ng/L, are displayed as boxplots for (A) M. loti, (B) S. meliloti, (C) R. leguminosarum, and (D) P. putida. n = 4 biological replicates. Supplementary Fig. 2. Assessing the B12 dependence of three lines of C. reinhardtii under different trophic conditions. The three lines include the ‘ancestral’ line prior to experimental evolution, ‘metE7’, a stable B12‐dependent line, and ‘revertant’, a B12 independent line that had reverted from a B12‐ dependent line. Cultures were grown heterotrophically (TAP medium in the dark), mixotrophically (TAP medium in continuous light), and photoautotrophically (Tris minimal medium in continuous light). B12 concentrations ranged from 0.5 to 512 ng·L−1 and precultures of the algae, which were grown with 200 ng·L−1 B12, were washed thrice and inoculated at a density of roughly 100 cells·ml−1 . (A) Cell density was measured by particle counter after 6 days of growth for mixotrophic cultures or 8 days for heterotrophic and photoautotrophic conditions. (B) Estimated maximal density of metE7 at unlimiting B12 concentrations calculated by fitting a Monod equation to data in panel A. (C) Estimated concentration of B12 required to produce half the maximal density of metE7 cells under each trophic condition calculated by fitting a Monod equation to data in panel A. n = 3–4, error bars = sd. Supplementary Fig. 3. Growth and B12 uptake of M. loti strains. The wildtype (MAFF303099) and B12 synthesis (BluB) mutant were grown in Tris minimal medium supplemented with 0.1% [file EMI-24-3134-s001.pdf]

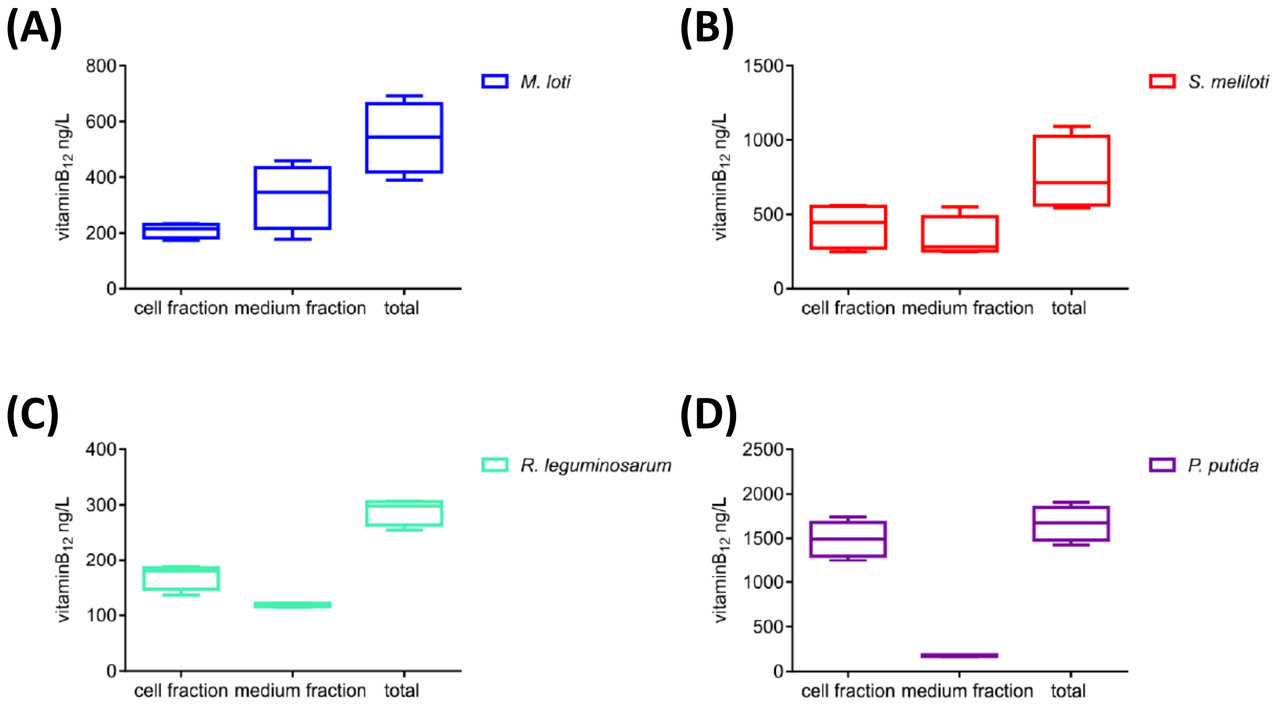

Supplementary Figure 1. Vitamin B_{12} levels in the cell and media fraction of axenic cultures of four B_{12}-producing bacterial strains. Bacteria were grown in TP medium + 0.1% glycerol with illumination in a 12:12 hour light:dark period at 100 $\mu E \cdot m^{-2} \cdot s^{-1}$ and 25°C with rotational shaking at 120 rpm. After 6 days of growth, cultures were collected, centrifuged, and the pellet (cell fraction) and supernatant (medium fraction) separated and their B_{12} content measured. The B_{12} concentrations, in ng/L, are displayed as boxplots for **(A)** *Mesorhizobium. loti*, **(B)** *Sinorhizobium meliloti*, **(C)** *Rhizobium leguminosarum*, and **(D)** *Pseudomonas putida.* n=4 biological replicates.

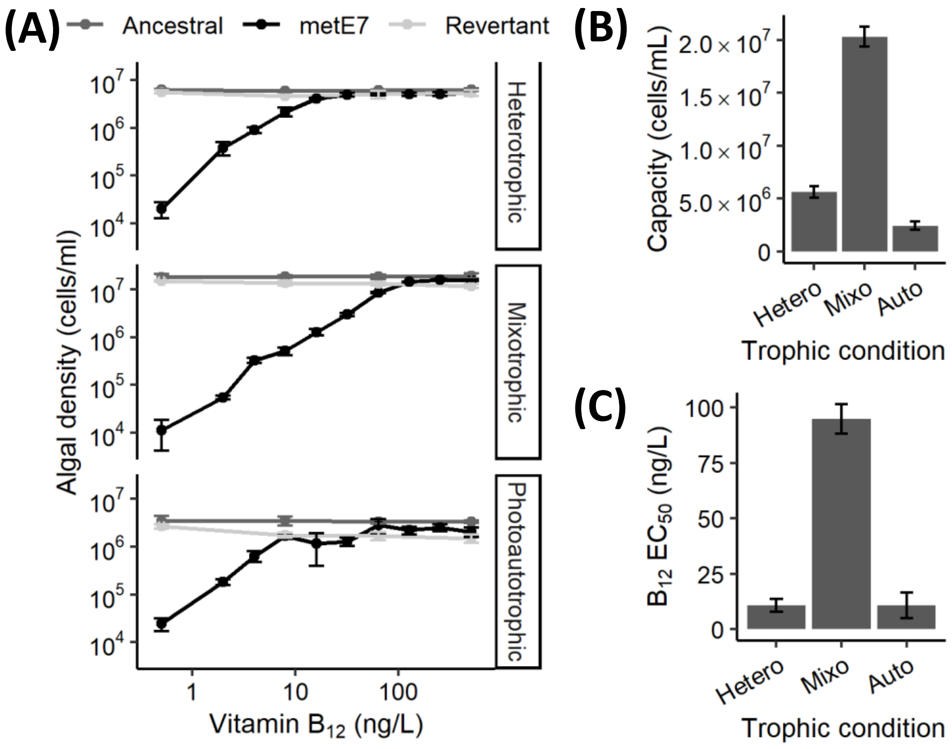

Supplementary Figure 2. Assessing the B_{12} dependence of three lines of *C. reinhardtii* under different trophic conditions. The three lines include the 'ancestral' line prior to experimental evolution, 'metE7', a stable B_{12}-dependent line, and 'revertant', a B_{12} independent line that had reverted from a B_{12}-dependent line. Cultures were grown heterotrophically (TAP medium in the dark), mixotrophically (TAP medium in continuous light), and photoautotrophically (Tris minimal medium in continuous light). B_{12} concentrations ranged from 0.5 to 512 $ng{\cdot}L^{-1}$ and precultures of the algae, which were grown with 200 $ng{\cdot}L^{-1}$ B_{12}, were washed thrice and inoculated at a density of roughly 100 $cells{\cdot}mL^{-1}$. **(A)** Cell density was measured by particle counter after 6 days of growth for mixotrophic cultures or 8 days for heterotrophic and photoautotrophic conditions. **(B)** Estimated maximal density of metE7 at unlimiting B_{12} concentrations calculated by fitting a Monod equation to data in panel A. **(C)** Estimated concentration of B_{12} required to produce half the maximal density of metE7 cells under each trophic condition calculated by fitting a Monod equation to data in panel A. n=3-4, error bars = sd.

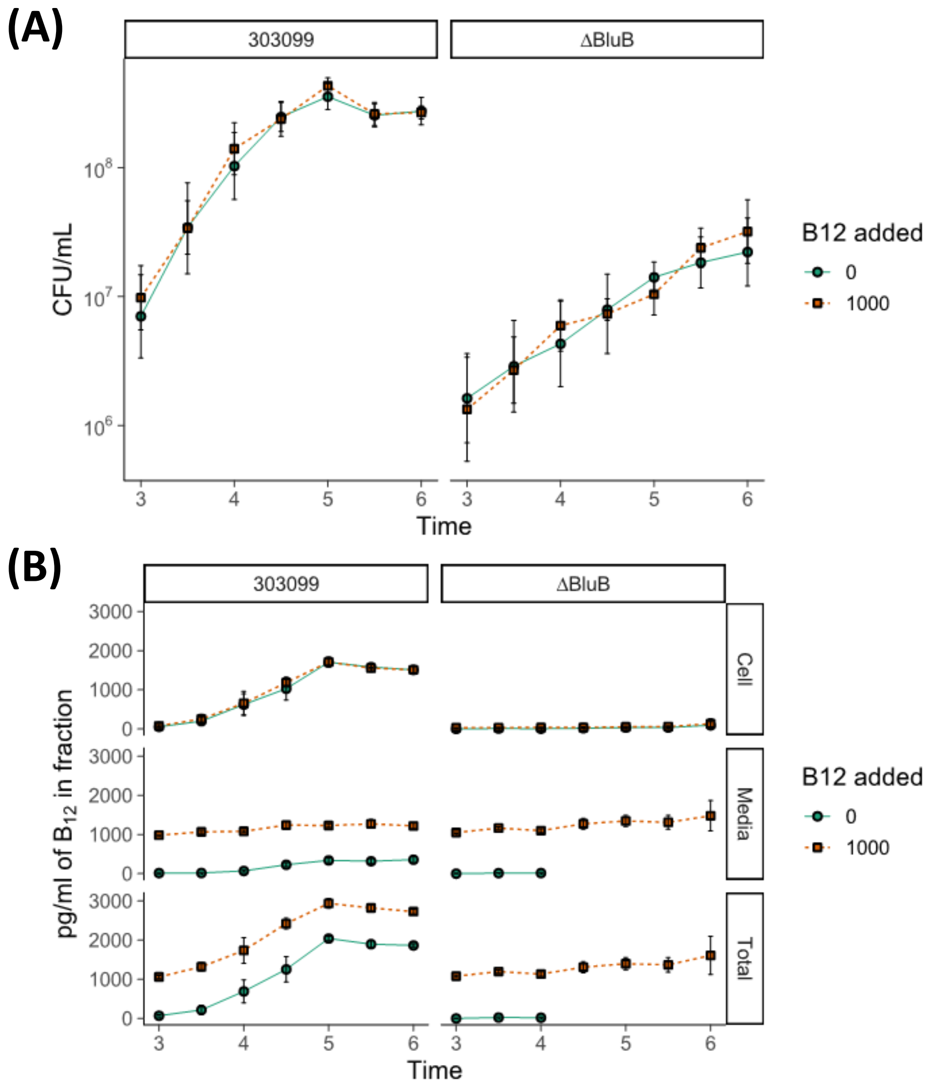

Supplementary Figure 3. Growth and B_{12} uptake of *M. loti* strains. The wildtype (MAFF303099) and B_{12} synthesis (BluB) mutant were grown in Tris minimal medium supplemented with 0.1% glycerol at 100 $\mu E \cdot m^{-2} \cdot s^{-1}$, and at a temperature of 25°C, with rotational shaking at 120 rpm over a period of 6 days with (1000 $ng \cdot L^{-1}$) or without added B_{12}. **(A)** Viable cells (colony forming units) of *M. loti* 303099 increased more quickly than the BluB mutant, but there was no significant effect of B_{12} on growth rate of either strain. **(B)** The addition of B_{12} had no effect on the B_{12} recovered in the cell fraction (top panel) indicating no B_{12} uptake. Instead, all the added B_{12} remained in the media (middle panel). Red lines = 1000 $ng \cdot L^{-1}$ of added B_{12}, Blue lines = no added B_{12}, Error bars = sd, n=4.

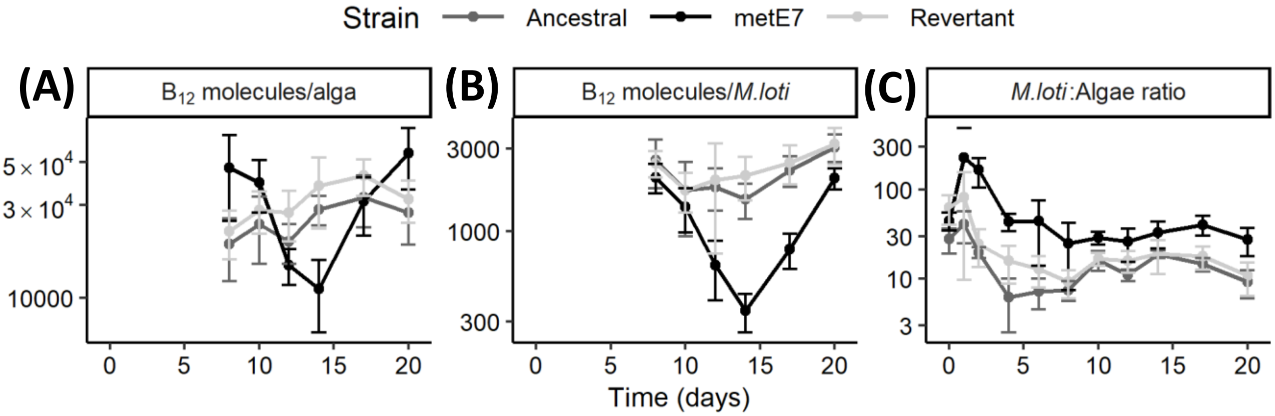

Supplementary Figure 4. Dynamics of the ratios of B_{12}, bacterial density and algal density during cocultures of *M. loti* and three strains of *C. reinhardtii* **(A)** B_{12} concentration expressed as molecules of B_{12} per algal cell reveal very similar levels although different dynamics for the three *C. reinhardtii* strains **(B)** B_{12} concentration expressed as molecules of B_{12} per *M. loti* cell reveal lower production in coculture with metE7 particularly around day 14 of coculture. **(C)** Bacteria:algae ratio was consistently higher in the metE7 coculture. Error bars = sd, n=5.

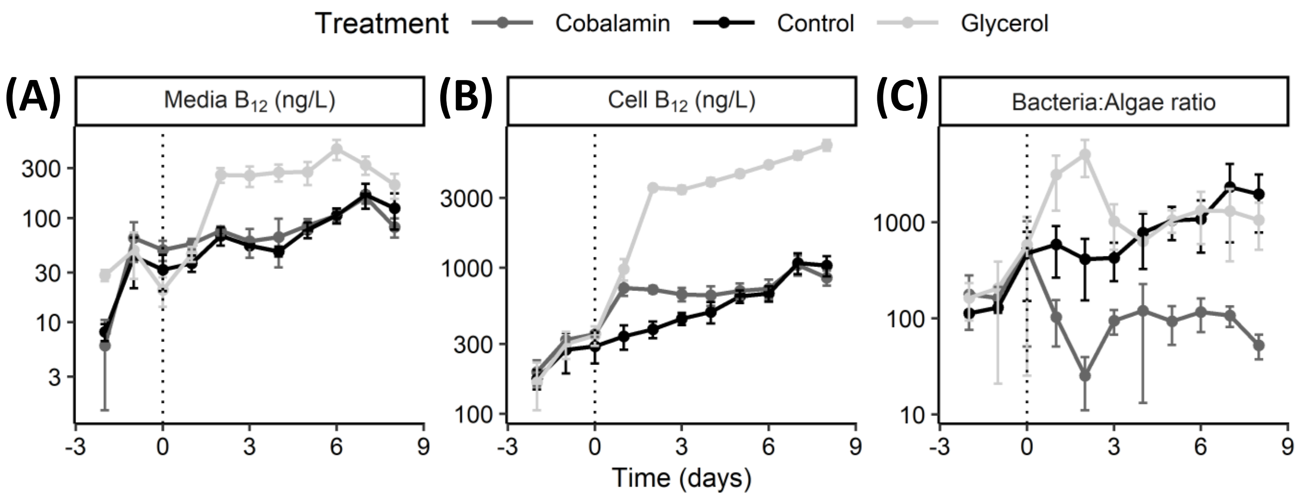

Supplementary Figure 5. Dynamics of B_{12} concentrations in the cellular and media fractions and bacteria:algae ratio in metE7+*M. loti* cocultures perturbed by nutrient addition. **(A)** B_{12} concentration in the media of cocultures reveals that the highest levels were found following addition of glycerol. **(B)** B_{12} concentration in the cellular fraction reveals that glycerol addition caused significantly higher B_{12} production. **(C)** Bacteria:algae ratio initially diverged after addition of glycerol or B_{12} followed by a smaller convergence. Error bars = sd, n=4.

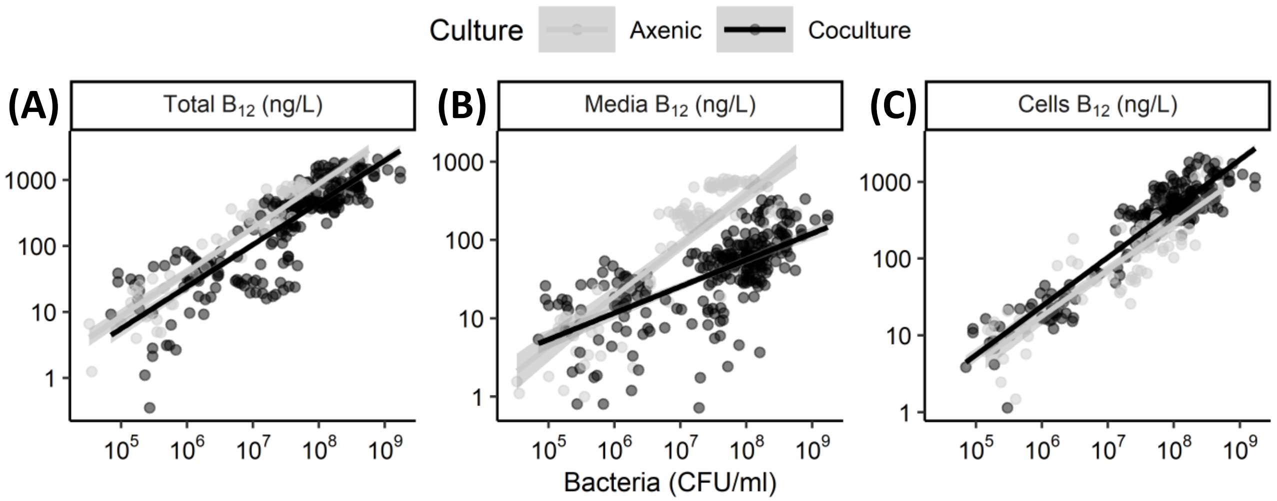

Supplementary Figure 6. *M. loti* does not increase B_{12} production in the presence of metE7. Several axenic cultures of *M. loti* with supplemented glycerol and cocultures containing *M. loti* and metE7 (without glycerol) were grown in TP medium at 25°C with illumination at 100 $\mu E \cdot m^{-2} \cdot s^{-1}$ over a 16:8 hour light:dark cycle for up to 32 days or up until the cultures crashed. B_{12} measurements of the media and cell fraction were made periodically **(A)** Total B_{12} is higher in axenic *M. loti* culture than coculture at the same *M. loti* density ($p<0.001$) **(B)** B_{12} in the media is significantly lower in cocultures than axenic cultures at high *M. loti* densities ($p<0.001$). **(C)** Cellular B_{12} is significantly higher in coculture than axenic culture at the same *M. loti* densities ($p<0.001$). Grey = *M. loti* axenic culture, black=metE7 + *M. loti* coculture. N(axenic)=106, N(coculture)=284, grey shaded region = 95% confidence interval.

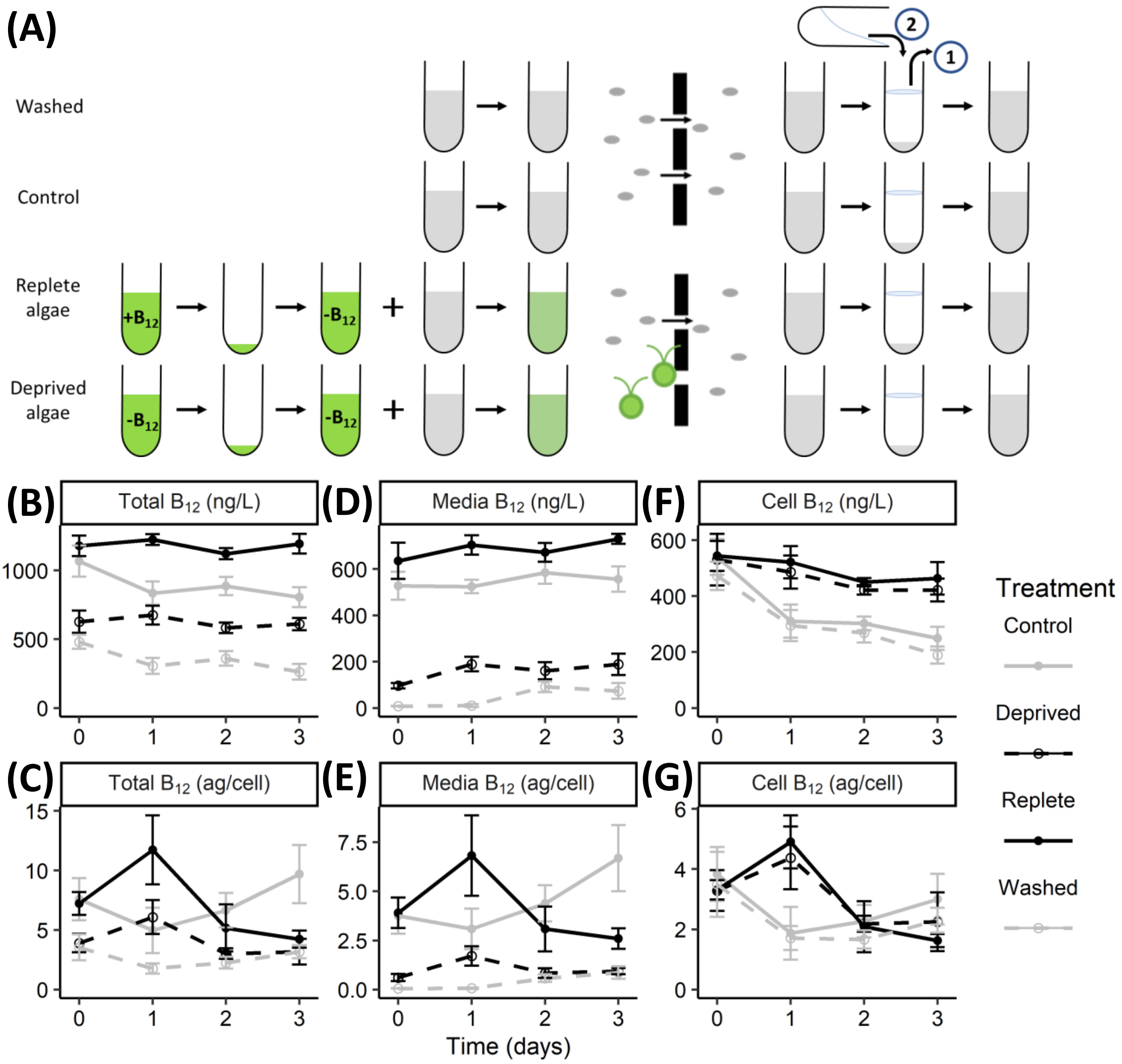

Supplementary Figure 7. B_{12} production by *M. loti* following removal of B_{12} from the culture media. **(A)** Experimental setup: Two sets of axenic *M. loti* cultures (grey) were inoculated with metE7 cells that were either saturated with (black solid) or starved (black dashed) of B_{12} and incubated for 1 hour. All 4 cultures were then passed through a 5 µm filter, removing all metE7 cells but not *M. loti*. These *M. loti* cultures were centrifuged, and the supernatant replaced with fresh Tris-min media in treatment 'washed' (grey dashed), or otherwise resuspended without replacing the supernatant (grey solid). The resuspended, newly axenic *M. loti* cultures were grown for 3 days with illumination in a 16:8 hour period at 100 $\mu E \cdot m^{-2} \cdot s^{-1}$ and 25°C with rotational shaking at 120 rpm. **(B)** Total B_{12} concentration in the culture, and **(C)** Total B_{12} per *M. loti* cell. **(D)** B_{12} concentration in the supernatant after centrifuging an aliquot of the sample, and **(E)** media B_{12} per *M. loti* cell. **(F)** B_{12} concentration in the cell pellet after centrifuging an aliquot of the sample, and **(G)** cell B_{12} per *M. loti* cell. Error bars = sd, n=4.

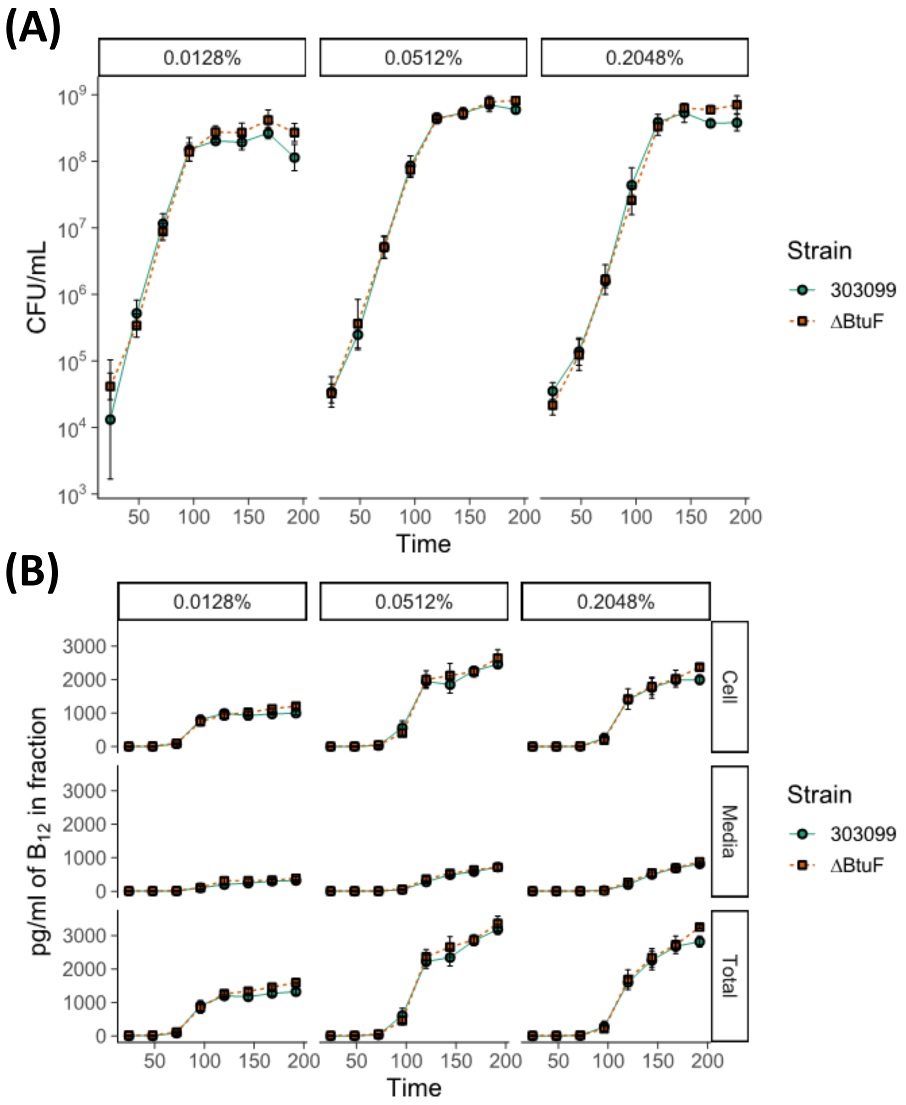

Supplementary Figure 8. Growth and B_{12} release of *M. loti* strains. The wildtype (MAFF303099) and B_{12} transporter (BtuF) mutant were grown in Tris minimal medium supplemented with various concentrations of glycerol at 100 μE·m^{-2}·s^{-1}, and at a temperature of 25°C with rotational shaking at 120 rpm over a period of 8 days. **(A)** Viable cells (colony forming units) of *M. loti* 303099 increased over time at the same rate as the BtuF mutant and both strains showed improved growth on increasing the glycerol concentration from 0.0128% (v/v) to 0.0512%, but not with a higher concentration. **(B)** The amount of B_{12} produced in the cells (top panel) and released into the media (middle panel) were not significantly different in the two strains, but as with the cell growth, did increase with the two higher glycerol concentrations. Blue lines = wildtype (MAFF303099), Blue lines = BtuF mutant, Error bars = sd, n=4.
